# Supplementary figures and images for: SPINT1 Expressed in Epithelial Cells of Choroid Plexus in Human and Mouse Brains
Source: Int J Mol Sci. 2025 May 27;26(11):5130. doi: 10.3390/ijms26115130 (PMC12155093; doi:10.3390/ijms26115130)

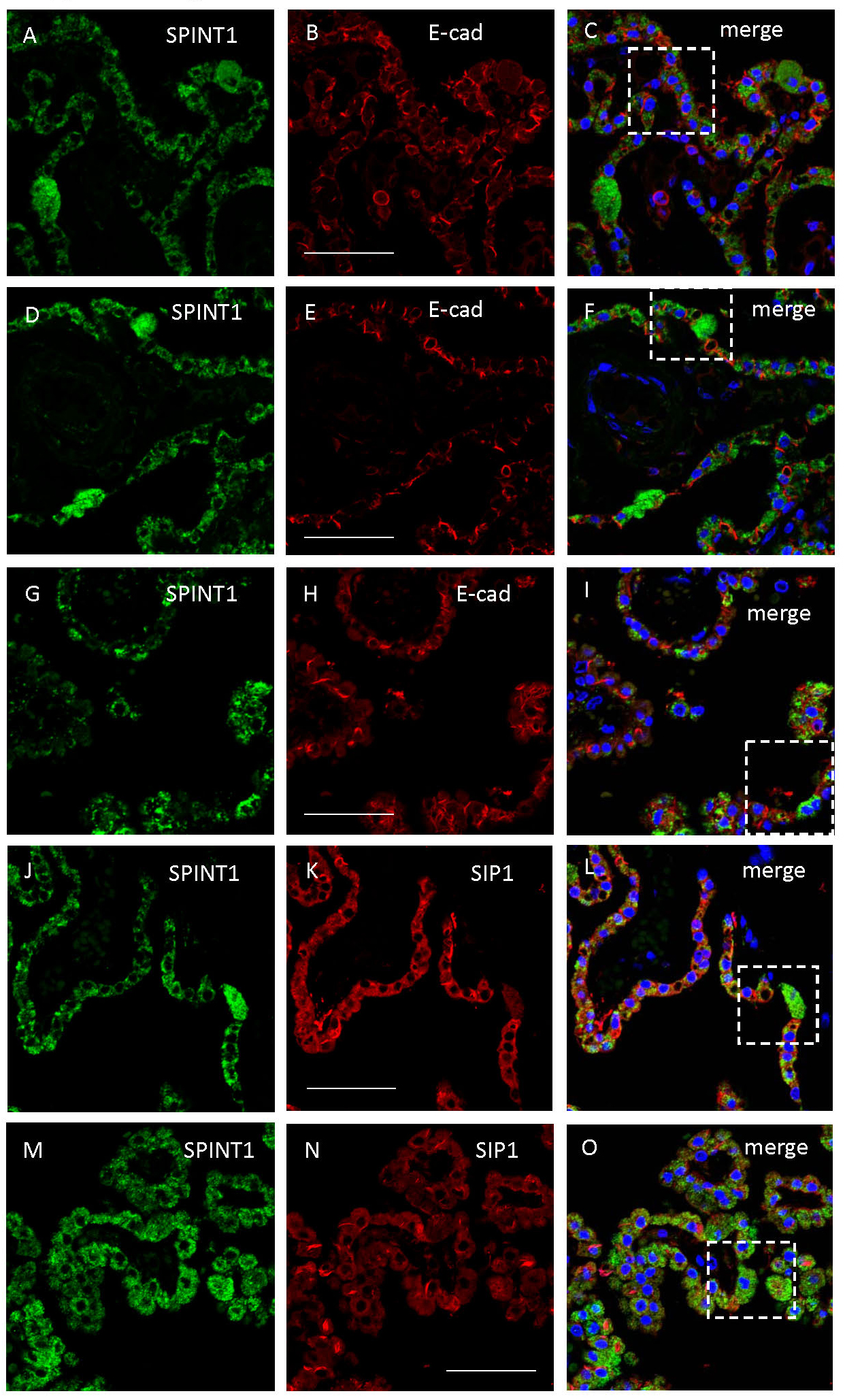

Supplement: Supplementary file 1 [file ijms-26-05130-s001.zip › 2025-SPINT1-r1-Figure S1.jpg]

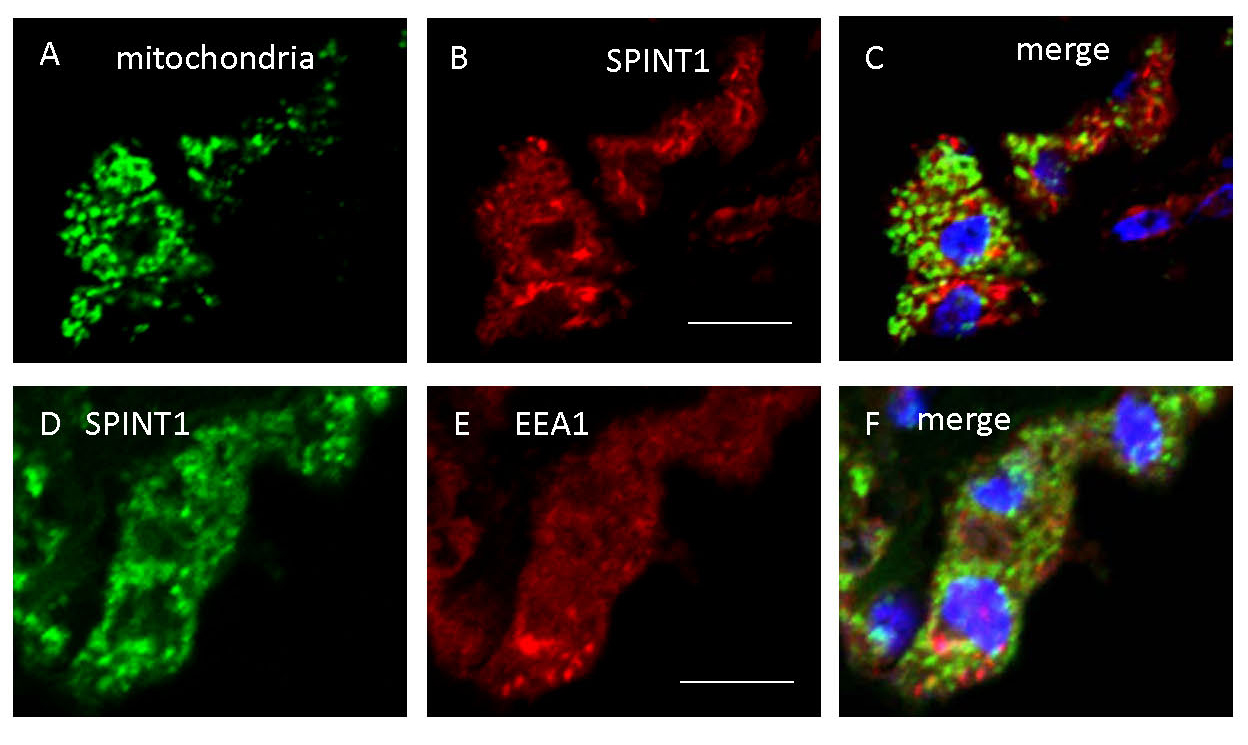

Supplement: Supplementary file 1 [file ijms-26-05130-s001.zip › 2025-SPINT1-r1-Figure S2.jpg]

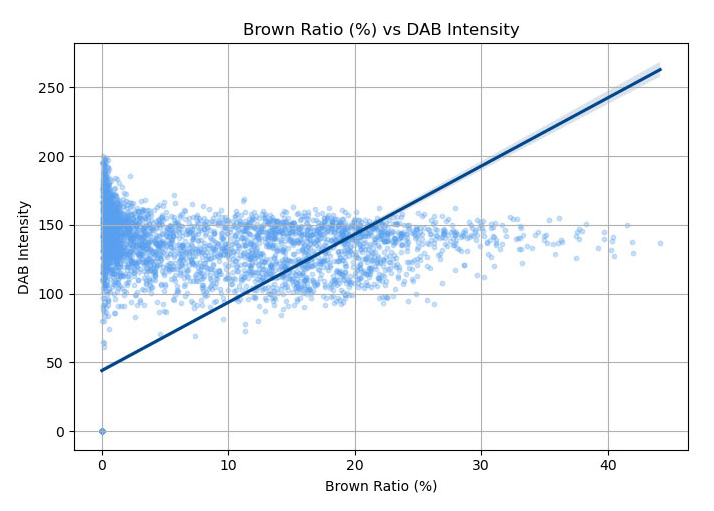

Supplement: Supplementary file 1 [file ijms-26-05130-s001.zip › 2025-SPINT1-r1-Figure S3.jpg]
